# Evidence for a K_ATP_ Channel in Rough Endoplasmic Reticulum (rerK_ATP_ Channel) of Rat Hepatocytes

**DOI:** 10.1371/journal.pone.0125798

**Published:** 2015-05-07

**Authors:** Sajjad Salari, Maedeh Ghasemi, Javad Fahanik-Babaei, Reza Saghiri, Remy Sauve, Afsaneh Eliassi

**Affiliations:** 1 Neurophysiology Research Center, Shahid Beheshti University of Medical Sciences, Tehran, Iran; 2 Department of Physiology, Medical School, Shahid Beheshti University of Medical Sciences, Tehran, Iran; 3 Neuroscience Research Center, Shahid Beheshti University of Medical Sciences, Tehran, Iran; 4 Department of Biochemistry, Pasteur Institute of Iran, Tehran, Iran; 5 Department of Molecular and Integrative Physiology and Membrane Protein Research Group, Université de Montréal, Montréal, Québec H3C 3J7, Canada; Cinvestav-IPN, MEXICO

## Abstract

We report in a previous study the presence of a large conductance K^+^ channel in the membrane of rough endoplasmic reticulum (RER) from rat hepatocytes incorporated into lipid bilayers. Channel activity in this case was found to decrease in presence of ATP 100 µM on the cytoplasmic side and was totally inhibited at ATP concentrations greater than 0.25 mM. Although such features would be compatible with the presence of a K_ATP_ channel in the RER, recent data obtained from a brain mitochondrial inner membrane preparation have provided evidence for a Maxi-K channel which could also be blocked by ATP within the mM concentration range. A series of channel incorporation experiments was thus undertaken to determine if the ATP-sensitive channel originally observed in the RER corresponds to K_ATP_ channel. Our results indicate that the gating and permeation properties of this channel are unaffected by the addition of 800 nM charybdotoxin and 1 µM iberiotoxin, but appeared sensitive to 10 mM TEA and 2.5 mM ATP. Furthermore, adding 100 µM glibenclamide at positive potentials and 400 µM tolbutamide at negative or positive voltages caused a strong inhibition of channel activity. Finally Western blot analyses provided evidence for Kir6.2, SUR1 and/or SUR2B, and SUR2A expression in our RER fractions. It was concluded on the basis of these observations that the channel previously characterized in RER membranes corresponds to K_ATP_, suggesting that opening of this channel may enhance Ca^2+^ releases, alter the dynamics of the Ca^2+^ transient and prevent accumulation of Ca^2+^ in the ER during Ca^2+^ overload.

## Introduction

Ion channels are present in endomembranes. These channels are thought to play an important role in cellular processes such as compensation for electrical charges, generation of a pH gradient [1], oxidative stress production [2, 3], and cell volume regulation.

Potassium channels have also been found in endo/sarcoplasmic reticulum membranes. Evidence for a K^+^ selective channel of high conductance was provided by Picard et al. (2002) [4] using sarcoplasmic reticulum (SR) membrane preparations from human (193 pS) and sheep (185 pS) atrial cells. The gating behaviour and permeation properties of this channel were unaffected by the addition of 4-AP and iberiotoxin (IbTx; a specific blocker of Maxi-K_Ca_ channels) [4]. Voltage-gated potassium channels were also identified in the SR of diaphragm [5] and frog skeletal muscle [6]. In diaphragm SR the channel conductance and gating properties were not affected by physiological concentrations of Ca^2+^, Mg^2+^, and the channel appeared insensitive to glyburide (a selective blocker of ATP-sensitive K+ channels), and charybdotoxin (ChTx) (blocker of Ca^2+^-activated K^+^ channels) [5]. It was proposed on the basis of these observations that SR K^+^ channels could serve in maintaining the Ca^2+^ homeostasis [5, 7].

In contrast to the SR, where voltage gated potassium channels have clearly been identified, there is little evidence for voltage activated K^+^ selective channels in the rough endoplasmic reticulum (RER) of non-excitable cells such as hepatocytes. We have shown in a previous work that the RER membrane of hepatocytes contains a large conductance (509 pS) K^+^ channel activated by voltage and inhibited by 4-AP (5 mM) [8]. Notably channel activity was decreased in presence of ATP 100 μM on the cytoplasmic side and totally inhibited at ATP concentrations greater than 0.25 mM [9]. These observations argue for the potential presence of a K_ATP_ channel in the RER membranes of hepatocytes. It was reported however that the activity of several Maxi-K_Ca_ channels could be altered by intracellular ATP [10]. Of particular interest is the observation that the Maxi-K_Ca_ channel we identified in a mitochondrial inner membrane preparation from rat brain was inhibited by mM ATP [11]. These results thus raise the possibility for the presence in internal organelles, including RER, of large conductance K^+^ channels sensitive to ATP not corresponding to K_ATP_ type channels. A study was thus undertaken to determine the nature of the ATP sensitive channel we identified in RER of hepatocytes. Our results indicated that the large conductance channels we characterized in RER of hepatocytes correspond to a K_ATP_ channel.

## Materials and Methods

### Ethical statement

All experiments were executed in accordance with the Guide for Care and Use of Laboratory Animals (National Institute of Health Publication No.80-23, revised 1996), as approved by the Research and Ethics Committee of Shahid Beheshti University of Medical Sciences (1100/1-87/11/21; 2008). Male Wister rats (180–210 g) were housed in a controlled environment (temperature 22 ± 2 _C, humidity 50 ± 10% and 12:12-h light/dark cycle) and were acclimatized for a week before use in experiments. Rats had free access to food and water ad libitum.

### Materials

L-a-phosphatidylcholine (L-a-lecithin) was extracted from fresh egg yolk as previously described [12]. 4-(2-hydroxyethyl)-1-piperazine ethane sulfonic acid (HEPES), 2-Amino-2-(hydroxymethyl)-1,3-propanediol (Trisma base), potassium chloride, tetraethyl ammonium (TEA), ChTx, IbTx, ATP, glibenclamide, tolbutamide and 4-AP were purchased from Sigma (St Louis, MO, USA) and n-decane was obtained from Merck (Darmstadt, Germany).

### Preparation of membrane vesicles

Rough microsomes derived from RER of rat liver cells were prepared as previously described [13, 14]. Briefly, three rats were anesthetised and euthanized by decapitation and then, livers were rapidly removed and homogenized in 30 ml of an ice-cold sucrose (0.25 M) solution at 2850 RPM using potter homogenizer (stage 1 for Western blotting). After adding 60 ml of ice-cold sucrose (0.25 M) solution, the homogenate was centrifuged at 8700 ×g for 13 min (stage 2 for Western blotting). Thereafter, the supernatant was centrifuged at 110000 ×g for 60 min at 4°C (Beckman model J-21B, USA). After dissolving the pellet in 9 ml of ice-cold sucrose 2 M, the solution was transferred to a 30 ml glass homogenizer, and was manually homogenized 8–10 times to obtain a homogenous suspension. The suspension was subsequently centrifuged at 300000 ×g for 60 min in a sucrose gradient, and the resulting pellet dissolved in 20 ml of sucrose 0.25 mM + imidazole 3 mM + Na pyrophosphate 0.5 mM (stage 3 for Western blotting), and centrifuged three times at 140000 ×g for 40 min. The resulting pellet (RER microsomes) was dissolved in 1 ml sucrose 0.25 mM + imidazole 3 mM at a final concentration of 7 mg/ml (stage 4 for Western blotting). Rough microsomes were stored in 10 μl aliquots in 250 mM sucrose/3 mM imidazole, pH 7.4 at -80°C for one month.

### Immunoblot analysis

Protein samples (35 μg) from each fraction were subjected to SDS-PAGE (n = 3) blotted and probed with antibodies directed against specific marker proteins: cox1 (Santa Cruz, SC-58347; Santa Cruz Biotechnology Inc., Heidelberg, Germany), actin (Santa Cruz, SC-1615), calnexin (Santa Cruz, SC-11397), 58 K Golgi protein (abcam, ab6284, Cambridge, UK)), SUR1 (Santa Cruz, SC-5789), SUR2A and SUR2B s (Santa Cruz, SC-32462 and SC-5793, respectively) and Kir6.2 (Santa Cruz, SC-11228; Santa Cruz Biotechnology Inc., Heidelberg, Germany). Expressions of the BK_Ca_ channel α-subunit in rat hepatocyte endoplasmic reticulum was determined using an antibody directed against BK_Ca_ channel α-subunit (rabbit polyclonal antibody, AB-104467, Abcam, Cambridge, UK). Secondary antibodies linked to horseradish peroxidase were obtained from GE-Biosciences. Blots were finally treated with ECL kit for chemiluminescence detection.

### Bilayer formation and vesicle fusion

Planar phospholipid bilayers were formed in a 300 μm-diameter hole. The cis (cytoplasmic side) and trans (luminal side) chambers held 4 ml of KCl 200 and 50 mM (pH 7.4), respectively. Planar phospholipid bilayers were painted using a suspension of L-a-lecithin in decane (25 mg/ml). Fusion of the vesicles was initiated mechanically by gently touching the bilayer from the cis side.

### Recording instrumentation and statistical analysis

Single channel currents were measured with a BC-525D amplifier (Warner Instrument, USA). The cis chamber was voltage-clamped relative to the trans chamber, which was grounded. Electrical connections were made by using Ag/AgCl electrodes and agar salt bridges. All recordings were filtered at 1 kHz digitized at a sampling rate of 10 kHz and stored on a personal computer for off line analysis using PClamp9 (Axon Instruments Inc, USA). Unitary channel conductance was calculated from the current—voltage relationship. Open channel probability (P_o_) was calculated using the standard event detection algorithms in PClamp9. P_o_ was calculated from segments of continuous recordings lasting 50 s. The significance of differences was determined by Student's t test. Data are expressed as mean ± S.E. (standard error).

## Results

The nature of the large conductance K^+^ channel we previously identified in the RER membrane of hepatocyte [8] was investigated in a series of experiments aimed first to characterize the channel pharmacological profile, and second to establish if the K_ATP_ Kir6.x and SUR subunits were present in our RER membrane preparation.

### Effect of a nonspecific K^+^ channel blocker, TEA^+^

**Fig 1:**
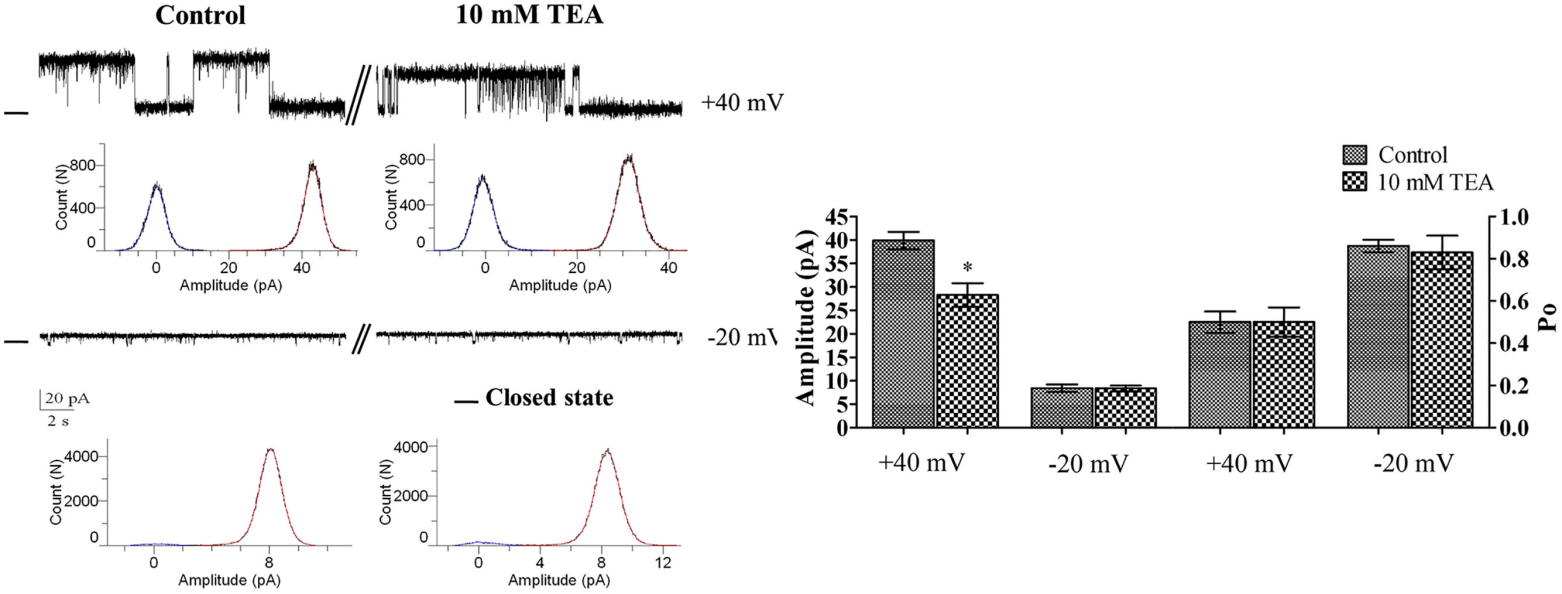
The effect of TEA^+^ on channel gating behavior at +40 and -20 mV. Examples of single channel recordings in controlled conditions (200/50 mMKCl;cis/trans) and immediately after cis addition of TEA^+^ 10 mM. Addition of TEA^+^ to the cis chamber caused a voltage dependent reduction in current jump amplitude that was significant at +40 mV. Results of these experiments are summarized in the bar graph shown in the right panel. Data are means ± S.E. (n = 3). The—shows the closed levels and * indicates P ≤ 0.05.

Fig 1 shows that the addition of 10 mM TEA^+^ to cis chamber significantly reduced the channel unitary current amplitude at positive potentials (Fig 1, n = 3). This result is compatible with the expected effect of a fast blocking agent.

### Effect of calcium ions and Ca^2+^-dependent K^+^ channels blockers, ChTx and IbTx

**Fig 2:**
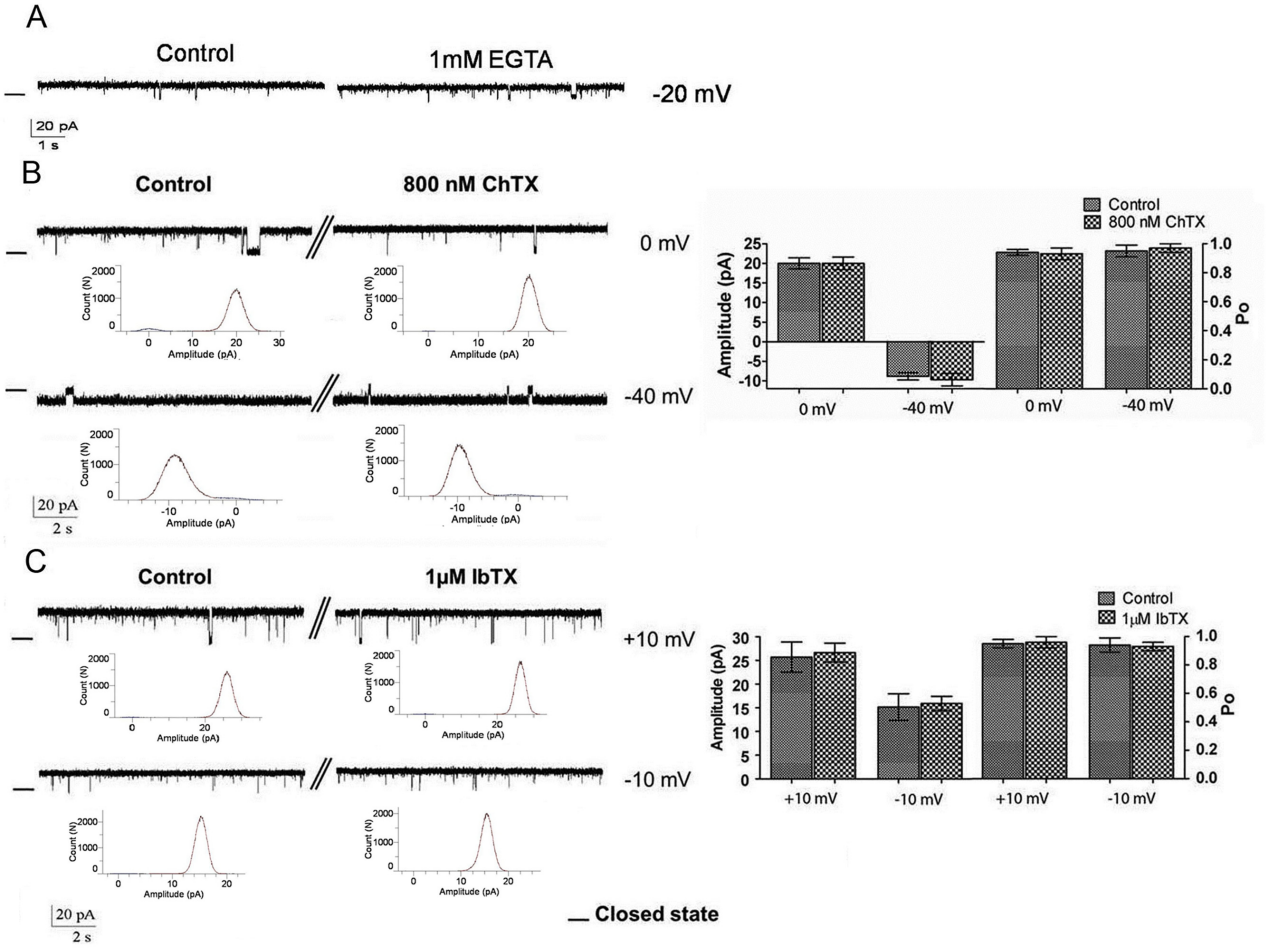
Effect of EGTA, charybdotoxin, and iberitoxin on channel activity. Representative unitary current recordings in control conditions (200/50 mMKCl; cis/trans) and after addition of 1 mM EGTA at -20 mV (A), 800 nM charybdotoxin (cis face) at 0 and -40 mV (B), and 1 μM iberiotoxin at +10 and -10 mV (C). Data are summarized in the bar graph presented in the right panel. There were no significant differences in open probability and current jump amplitude (n = 2, n = 4, n = 5, respectively), indicating that the K^+^ channel from RER does not correspond to a Max-K_Ca_ channel. The—indicates the closed levels.

We have already shown that the large conductance K^+^ channels in RER are inhibited by ATP in a dose-dependent manner [9]. Since, both Maxi-K_Ca_ and K_ATP_ channels are sensitive to ATP, we considered the possible effect of Ca^2+^ ions and Maxi-K_Ca_ blockers on the K^+^ channel from RER membranes. Fig 2A–2C show examples of single channel recordings obtained at -20 mV. Changes in channel activity were not observed after addition of 1 mM EGTA, arguing for the K^+^ channel we identified in the RER not being Ca^2+^ sensitive (Fig 2A). In addition, cis addition of IbTx (1 μM; n = 5), a specific inhibitor of Maxi-K_Ca_ channels, and ChTx (800 nM; n = 4) failed to modify the channel open probability and amplitude (Fig 2B and 2C). These results are summarized in the bar graph presented in the right panel. Altogether these observations provide evidence for K^+^ channel in RER not corresponding to a Max-K_Ca_ channel.

### Effect of sulfonylureas on channel activity

**Fig 3:**
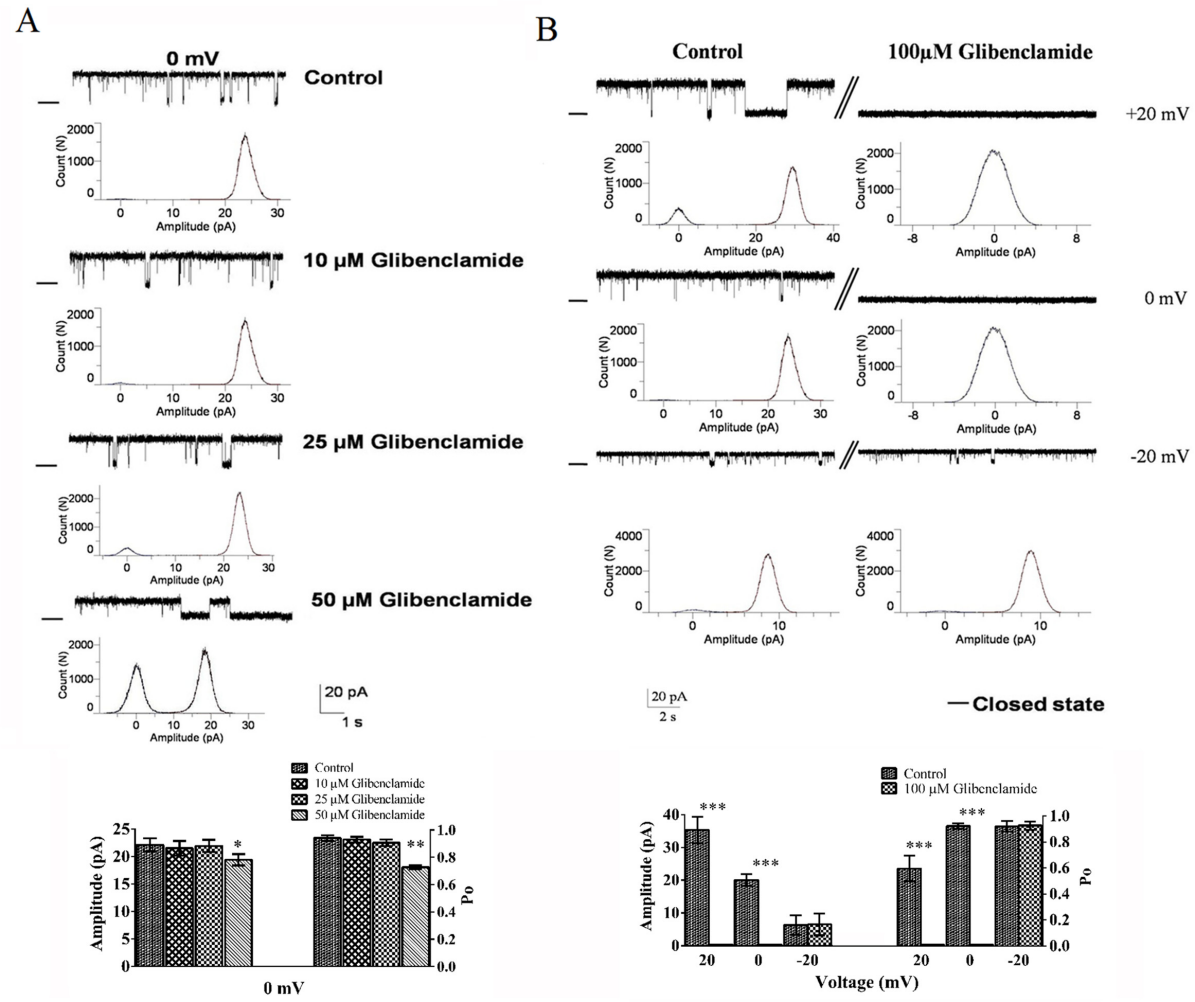
The effect of glibenclamide on channel activity at +20, 0 and -20 mV. Representative unitary current recordings in control conditions (200/50 mMKCl; cis/trans) and after cis addition of 10, 25, 50 (A) or 100 μM glibenclamide (B) at different voltages. Histogram bars show current amplitudes and P_o_ of reconstituted channels in absence or presence of glibenclamide. The addition of 50 μM glibenclamide resulted in a decreased current jump amplitude and a reduction in P_o_. 100 μM glibenclamide caused a total channel inhibition at +20 and 0 mV (n = 5), but was ineffective at -20 mV. The significant difference in the channel amplitude and P_o_ values are marked by asterisks (*P < 0.05 and ** P_o_ < 0.01, respectively, n = 5). Data are means ± S.E. Closed levels are indicated by –.

In additional experiments, the effect of glibenclamide and tolbutamide, two well-known sulfonylureas K_ATP_ channel blockers, was examined on RER channel activity. Fig 3A shows that the addition of 10 and 25 μM glibenclamide on the cis side had no significant effect on the channel Po and current jump amplitude at 0 mV (n = 2), while glibenclamide at 50 μM caused a small but significant decrease in the amplitude of the current jump and channel P_o_. In contrast a total block of cannel activity was observed with 100 μM glibenclamide in the cis chamber at positive but not negative potentials (Fig 3B). These results are summarized in the bar graph presented in the lower panel.

**Fig 4:**
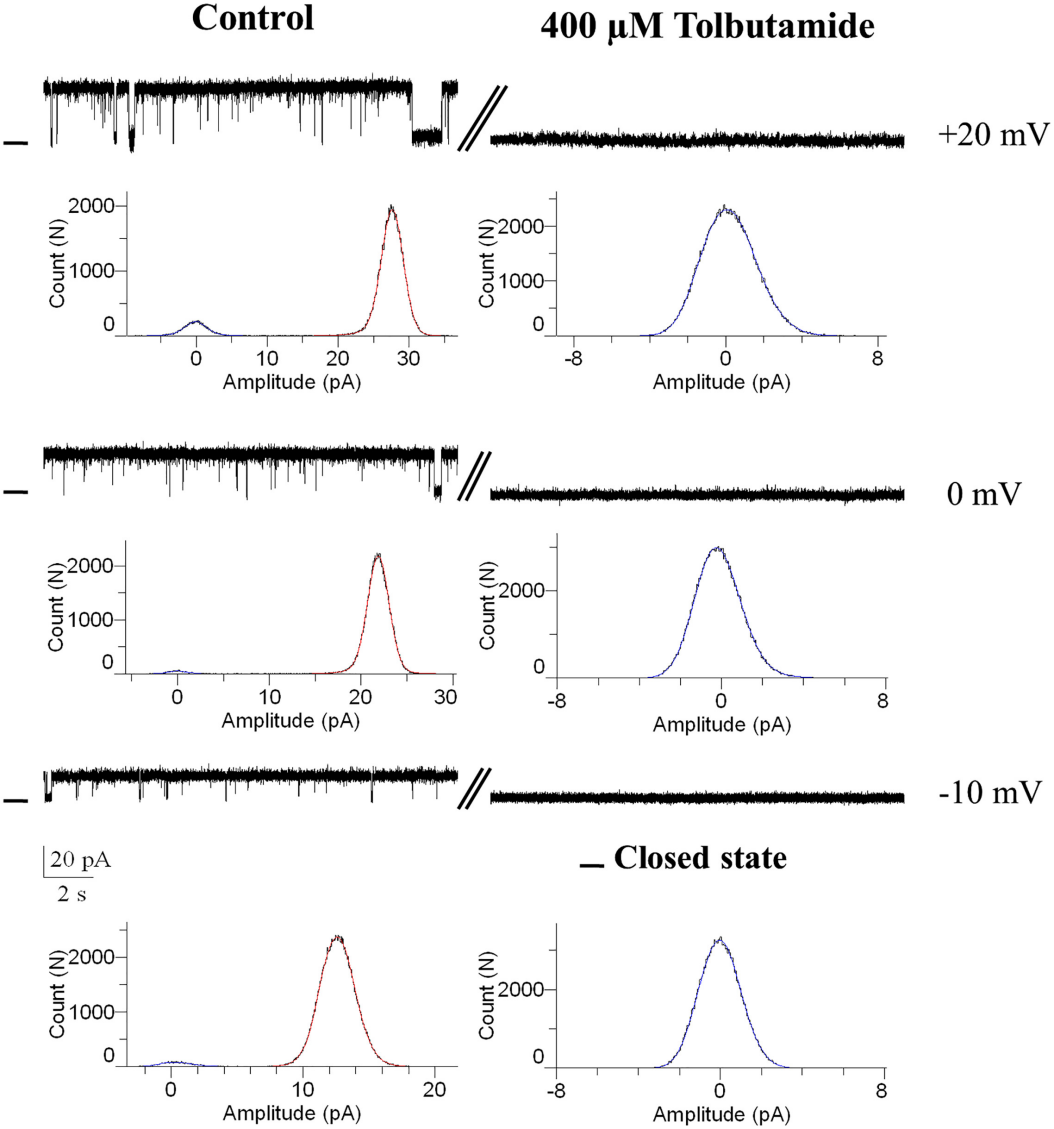
Effect of tolbutamide on channel gating behavior at +20, 0 and -10 mV. Examples of single channel recordings in control conditions (200/50 mMKCl; cis/trans), and immediately after cis addition of tolbutamide 400 μM (n = 5). Tolbutamide caused a total voltage independent inhibition of channel activity. The—indicates the closed levels.

Complementary to these observations the results presented in Fig 4 show that the K^+^ channel from RER was completely inhibited by addition of tolbutamide (400 μM) to the cis compartment at negative and positive potentials (n = 5). These observations argue for the K^+^ channel in RER having the characteristics of a K_ATP_ channel.

### Western blot analysis of K_ATP_ channel subunits

**Fig 5:**
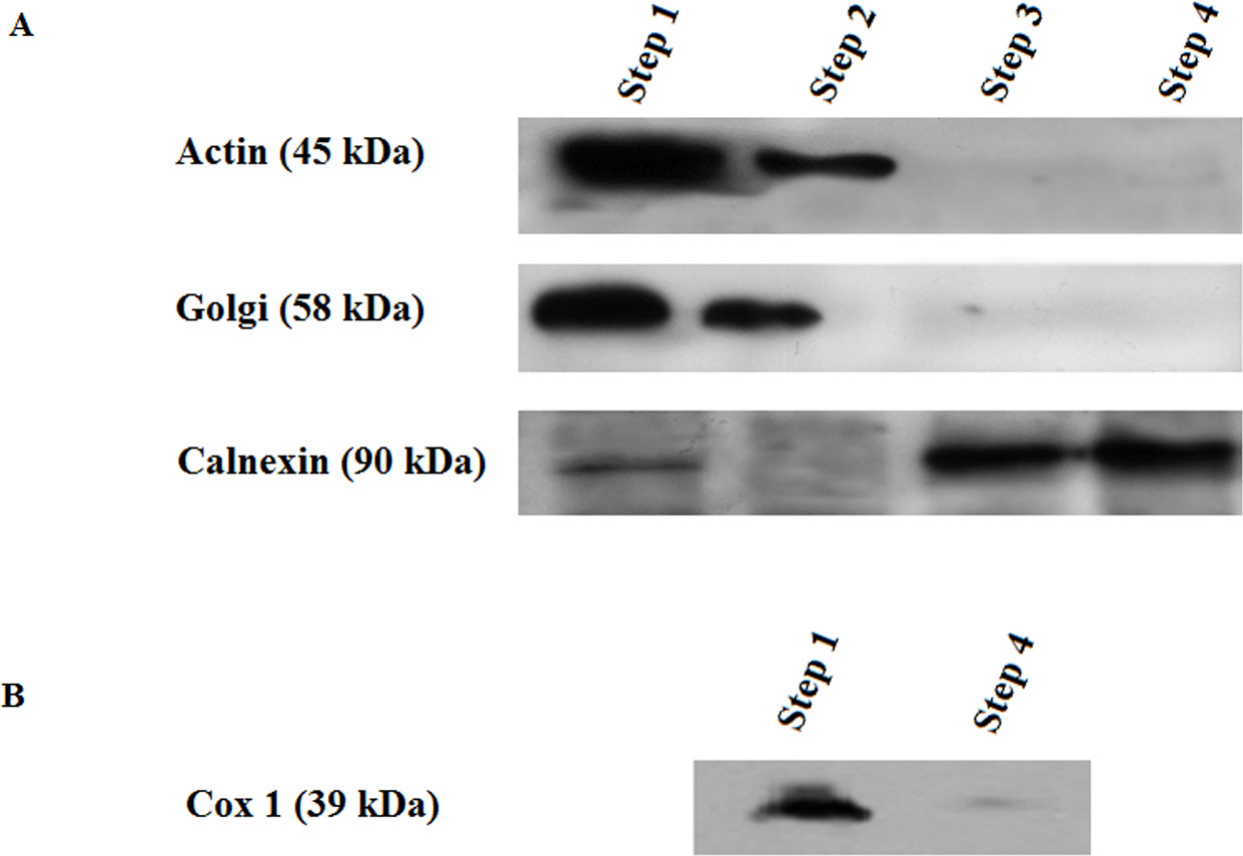
Characterization of the cell fraction used for channel incorporation. Membranes were probed with organelle specific antibodies. A) Plasma membrane marker: Actin (C-11), 45 kDa; Golgi marker: 58 K Goli protein; endoplasmic reticulum marker: Calnexin, 90 kDa, and in B) Mitochondrial membrane marker: Cox1 (1D6), 39 kDa. Although mitochondrial membranes were present in the homogenate (step 1) these membranes were removed through the purification procedure we used to obtain RER membranes (step 4). Steps 1 and 4 are described in the “methods and materials” section.

A Western blot analysis was next conducted to determine if K_ATP_ subunits were present in microsomes prepared from rat hepatocytes. We first evaluated the purity of our microsome preparation using antibodies directed against various cellular proteins considered unique to particular subcellular regions (Fig 5A). As seen in Fig 5B, the homogenate (step 1) contains as expected mitochondrial inner membrane the quantity of which was substantially reduced in final step (step 4) of RER membrane preparation.

**Fig 6:**
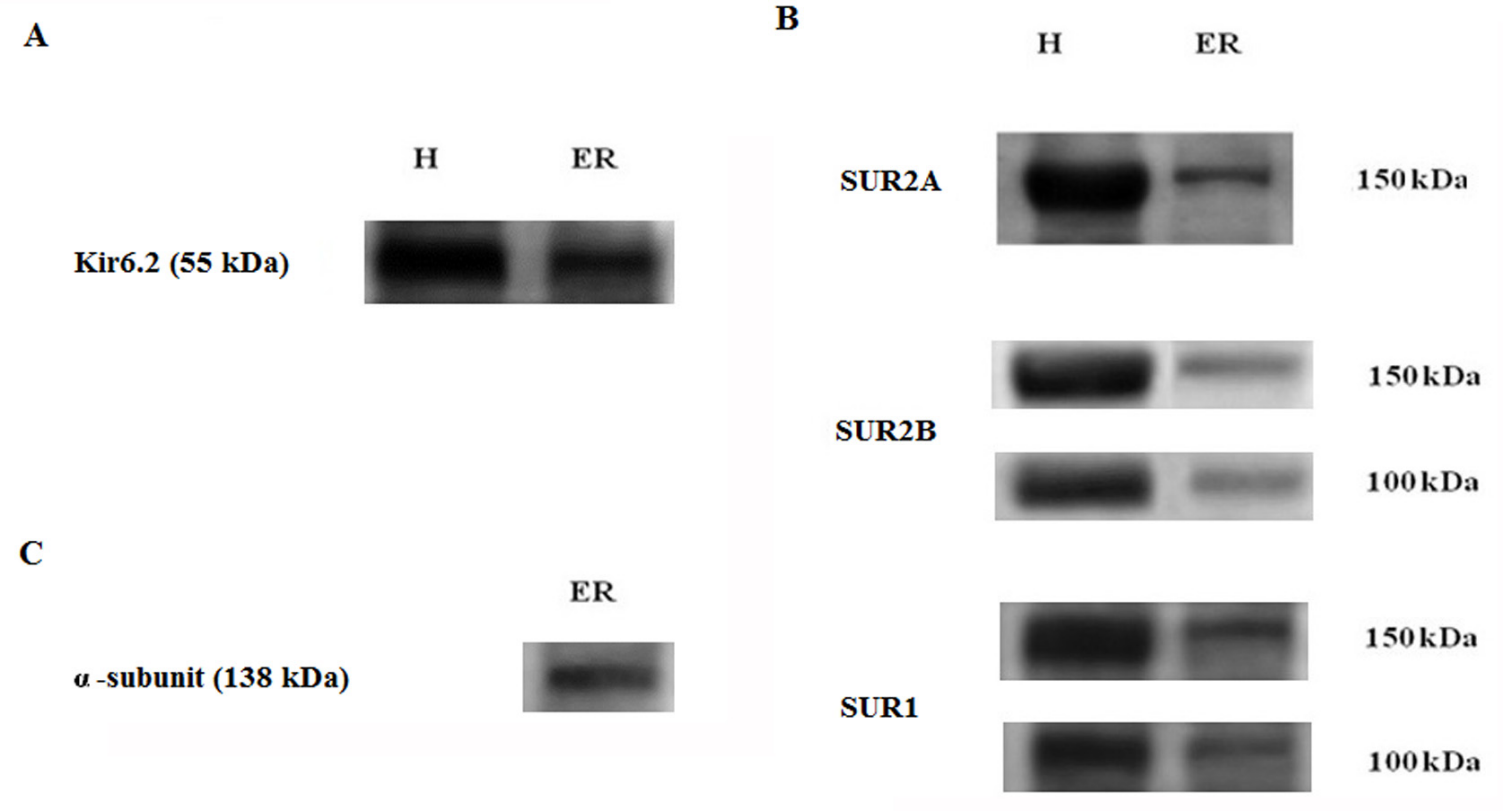
Western blot analysis of Kir6.2 and SURs in the rat microsome fractions. (A) The goat anti-Kir6.2 antibody led to the identification of a prominent ~55 kDa band in microsome fractions, a molecular weight compatible with a Kir6.2 K_ATP_ subunit. (B) ~150 kDa band was detected using the goat anti-SUR2A antibody, whereas the goat anti-human SUR2B and SUR1 antibodies led to the labeling of two bands of 150 and 100 kDa respectively. The 100 kDa may be resulting from proteolysis of the 150 kDa paptide. (C) Western blot indicating that of the Maxi-K_Ca_ channel α-subunit (138 kDa) is expressed in rat endoplasmic reticulum membranes.

We next probe for the presence of K_ATP_ subunits in our RER microsome preparations using antibodies directed against the K_ATP_ Kir6.2, SUR1, SUR2A, and SUR2B subunits. Labelling with the anti Kir6.2 antibody showed a specific band at 55 kDa, a molecular weight corresponding to the molecular weight estimated for Kir6.2 (Fig 6A) (n = 4). The results presented in Fig 6B indicate in addition that the anti-SUR2A antibody could labelled a 150 kDa band in crude and RER fractions, whereas two distinct bands at 100 kDa and 150 kDa were detected using the anti-SUR2B and anti-SUR1 antibodies in both the homogenate and RER membrane preparation respectively. These data confirm the presence of the K_ATP_ Kir6.2, SUR1 and SUR2 A and B subunits in the RER membrane fraction used for channel incorporation. We also tested for the presence of BK_Ca_ channel protein in the endoplasmic reticulum membrane [15, 16]. The immunoblot presented in Fig 6C indicates in this regard that the BK_Ca_ channel α subunits protein is expressed in our endoplasmic reticulum membrane preparation, despite the absence of detectable BK_Ca_ channel activity in our incorporation experiments.

## Discussion

K_ATP_ channels were originally discovered in the heart, and have since been found in numerous tissue preparations [17, 18]. K_ATP_ channels are also present in endomembranes, in particular in mitochondria (mitoK_ATP_) [19]. The main functional feature of K_ATP_ channels arises from their sensitivity to internal ATP levels, with K_ATP_ being blocked at submilimolar ATP concentrations. Internal block by ATP is not however exclusive to K_ATP_ channels. In fact, several studies have shown that Maxi-K_Ca_ channels could be inhibited by intracellular ATP [10, 20, 21]. In this regard we have presented evidence that a Maxi-K_Ca_ channel in the inner membrane of mitochondria could be blocked by internal ATP in the mM rage [11]. These observations casted some doubt on the actual nature of a K^+^ channel of large conductance we identified in a RER membrane preparation from hepatocyte as this channel could be inhibited by ATP within the same concentration rage [8, 9]. We demonstrated in this work through Western blotting analysis and pharmacological characterization that the K^+^ channel of large conductance identified in RER membranes possesses features associated to a K_ATP_ type channel

The K^+^ channel blocking agent TEA^+^ was first used to probe the rerK^+^ channel conduction pathway. TEA acts from both sides of the channels and its blocking action is voltage-dependent [22, 23]. Our results showed that 10 mM TEA^+^ decreased unitary current amplitude of the K^+^ channel from RER (RERK^+^) at positive voltages, a result compatible with a fast block mechanism. These results do not however enable one to discriminate between K_ATP_ and Maxi-K_Ca_ channels as TEA^+^ was found to block Maxi-K_Ca_ channels [20, 21], albeit at lower concentrations than those reported to block K_ATP_.

Several pharmacological blockers of Maxi-K_Ca_ channels are known, including the scorpion-derived peptides ChTx and IbTx [24, 25]. In the current study, we showed that addition of IbTx and ChTx to the cis side did not affect the channel activity, thus confirming that the channel does not belong to Maxi-K_Ca_ channel family. This conclusion is further supported by the observation that the channel remained active in zero Ca^2+^ conditions. Notably the high conductance Slack (‘sequence like a Ca^2^+-activated K^+^ channel; also termed Slo2.2) and Slik (also termed Slo2.1) [26], sodium-activated K^+^ (K_Na_) channels [27–29] have been documented to be insensitive to the Maxi-K_Ca_ channel blocking agent iberiotoxin but inhibited by TEA^+^ at the millimolar (10 mM) concentration range [26, 28]. More importantly, application of ATP to the cytoplasmic face of Slick channels was found to reduce channel activity [28]. These features are in line with the pharmacological profile we obtained for the K^+^ in RER membranes. It has been suggested that the Na^+^ concentration required for channel gating in both cases ranges from 7 mM to 180 mM [29]. As all our experiments were performed at contaminant levels of Na^+^ (≈ 100 μM), the possibility that the K^+^ channel from RER corresponds to either Slo2.1 or Slo2.2 can be ruled out. However observations by Kim et al. (2007) [30] indicated that K_Na_ channels could not be inhibited in the presence of a K^+^ channel blocker cocktail containing a mixture of Ba^2+^, glibenclamide, 4-AP, apamin, and quinidine [30], a result incompatible with the blocking effect of glibenclamide we observed on the K^+^ channel from RER membrane.

Sulfonylurea compounds are known to bind to the SUR subunit and inhibit the K_ATP_ channel activity [31, 32]. To determine if the endoplasmic reticulum potassium channel in rat hepatocytes corresponds to K_ATP_ channel, we tested the effects of a cis application of tolbutamide and glibenclamide on channel activity. Fig 4 demonstrates that the addition of 400 μM of tolbutamide to the cytoplasmic side (cis chamber) completely blocked channel activity. Furthermore, our result showed that glibenclamide at 100 μM inhibited the channel activity at positive potentials, but failed to affect the channel unitary current amplitude and open probability at negative potentials. The mechanism by which glibenclamide inhibits the ATP sensitive K^+^ channel in RER remains to be established. It has been suggested that glibenclamide was not blocking the channel directly but might act by binding either to the voltage gate and/or to the inner mouth of the channel leading to enhanced hydrophobic interactions between the two and a stabilization of the channel inactivated state [33]. Inhibition of channel activity by glibenclamide and tolbutamide was in the present work observed at micromolar concentrations. Notably, the K_ATP_ channel sensitivity to sulfonylurea derivatives was found to vary over a three orders of magnitude depending on the cell type. For instance K_ATP_ channels in pancreatic β-cells, cardiac muscle, and brain were found to be inhibited by glibenclamide at nanomolar concentrations, whereas K_ATP_ channels in other cells including epithelial cells appeared more glibenclamide resistant with concentrations for half- inhibition in the micromolar to millimolar ranges [34–36]. It has been suggested that the cytoskeleton affected K_ATP_ channel sensitivity to sulfonylureas inhibition, with a disruption of cytoskeleton structure by DNaseI leading to a 20 folds decrease in sulfonylureas affinities to K_ATP_ [37]. Because ER extraction involves disruption of the cell cytoskeleton it is possible that the low affinity we observed for glibenclamide and tolbutamide induced channel inhibition partly arised for the absence of cytoskeleton. Altogether, our pharmacological results would support the presence of a K_ATP_ channel in the hepatocytes RER membrane.

K_ATP_ channels are octomeric structures comprising four pore-forming Kir6.1 or Kir6.2 subunits and four sulfonylurea receptors (SURs) encoded by two distinct genes, SUR1 and SUR2 (SUR2A and SUR2B). Kir6.2 was reported to be expressed in ER fractions of pancreatic beta cells [38] and cardiomyocytes [39]. Our current Western blot results also argue for Kir6.2 being present in the RER membrane of hepatocytes and for the presence of the SUR1, SUR2A, and SUR2B subunits (Fig 6). Our Western blot results are unlikely to be explained by the presence of contaminants coming from other subcellular compartments, as we failed to detect the expression of plasma membrane or Golgi matrix specific proteins in our microsomal preparations. To our knowledge, this is the first study addressing the combination of SURs subunits in endoplasmic reticulum of rat hepatocytes.

There is evidence that the sensitivity of the channel to different sulfonylureas could depend on the SUR isoform. For instance, Kir6.2-SUR1 but not Kir6.2-SUR2A channels are blocked by tolbutamide with high affinity [40, 41]. Glibenclamide blocks both types of channels with high affinity. In line with these observations, reconstitution of K_ATP_ channels by co-expression of Kir6.2 and SUR2 in COS cells, and sulfonylurea binding measurements have revealed that despite similar structural features between SUR2 and SUR1, SUR2 binds glibenclamide with a lower affinity compared to SUR1, demonstrating that different responses to sulfonylureas may be linked to the SUR isoform [42]. These data, added to the fact that glibenclamide consists in a tolbutamide moiety plus a non-sulfonylurea meglitinide group known to inhibit K_ATP_ channels [43, 44] suggest that SUR1 contains a high-affinity tolbutamide- binding site missing in SUR2A. Our Western blot analysis provided unambiguous evidence for the presence of the Kir6.2 and SUR2A K_ATP_ subunits in the RER membrane of hepatocytes. Our analysis also showed two bands of 150 kDa and 100 kDa for the SUR2B and SUR1 subunits. It has been reported that the antiSUR2B anti-body recognises polypeptides of different molecular weights (150, 100, and 55 kDa) in some microsomal fractions including microsomes from rat heart sarcolemmal membranes [45]. More importantly, since the sequences of antigen peptide in SUR2B are highly homologous to the corresponding region in SUR1, we cannot currently rule out the possibility that the bands assigned to SUR2B can also be assigned to SUR1 [45]. Our results thus suggest that Kir6.2 and SUR2A are expressed in RER membrane of hepatocyte together with SUR2B and/or SUR1. There are supporting data that SUR1 and SUR2A coassemble readily and randomly to form heteromeric K_ATP_ channels [46]. Furthermore, Wheeler et al. demonstrated that SUR1, SUR2A, and SUR2B can coassemble in all the possible pair-wise combinations to form functional K_ATP_ channels with distinct properties, resulting in an enhanced functional and pharmacological diversity [46]. Determination of the Kir6.2 and SUR subunits exact combination will require further investigation.

Several groups [15, 16] including ours (present work) have provided evidence for the presence of the BK_Ca_ channel α-subunit (pore-forming subunit) in endoplasmic reticulum membrane. Notably channel activity could not be detected at single channel level in these preparations. These observations contrast with results obtained from mitochondria and nucleus membranes where BK_Ca_ channel activity has been documented [47,48, 11]. We cannot currently rule out the possibility of a non functional BK_Ca_ isofrom present in our RER membrane preparation. Confirmation of this proposal will await further work.

The precise functional role of K_ATP_ channels in RER is currently unknown. Both SR/ER Cl^-^ and K^+^ channels act as counter transport systems during rapid Ca^2+^ release and uptake as to keep electroneutrality and maintain the electrochemical driving force acting on Ca^2+^ ions [49, 50]. Given that Ca^2+^ regulations by the ER is prominent in cellular apoptosis [51, 52], these data suggest that K_ATP_ in RER membrane channel may contribute to the regulation of ER-mediated cellular mortality so that a defect in this regulatory process may cause a progressive loss of cell function and the generation of a cellular pathological state.
